# Supplementary material for: Anti-Oxidant and Anti-Aging Effects of Phlorizin Are Mediated by DAF-16-Induced Stress Response and Autophagy in Caenorhabditis elegans
Source: Antioxidants (Basel). 2022 Oct 8;11(10):1996. doi: 10.3390/antiox11101996 (PMC9598670; doi:10.3390/antiox11101996)
Supplement: Supplementary file 1 [file antioxidants-11-01996-s001.zip › antioxidants-1929608-supplementary.pdf]

Table S1: Primer set of each gene used for quantitative RT-PCR

Gene Name	Primer	Sequence
ama-1	forward	5'-CGG AGC AGC CAG GAA CTT C-3'
	reverse	5'-AAC GGG AAA AAT CTT ATG AAT-3'
skn-1	forward	5'-CTC TCT TCT GGC ATC CTC TAC CA-3'
	reverse	5'-TTC TTG GAT TCT TCT TCT TGT TCG T-3'
ctl-1	forward	5'-AAT GGA TAC GGA GCG CAT AC-3'
	reverse	5'-GCG TCA GTT GGA TCG AGA TT-3'
sod-3	forward	5'-TGG TGG TGG ACA CAT CAA TC-3'
	reverse	5'-ACC GAA GTC GCG CTT AAT AG-3'
gst-4	forward	5'-GCT GAA GCC AAC GAC TCC AT-3'
	reverse	5'-GAC CGA ATT GTT CTC CAT CGA-3'
bec-1	forward	5'-AGG AGC TGG AGC AAC AGT TGA AGA-3'
	reverse	5'-ATA TTG ACG TTC GGC TTC CAG CGA-3'
lgg-1	forward	5'-AAC AAC TTT GAG AAG CGT CGT GCC-3'
	reverse	5'-TCT TCT GGA CGA AGT TGG ATG CGT-3'

Table S2: Effect of phlorizin on lifespan in *C. elegans*

	**Phlorizin (μM)**	**Mean lifespan (d)**	***P* value[1)]**	**% effect[2)]**
1st experiment	0	17.5		
	10	20.6	< 0.001	18.0
2nd experiment	0	18.4		
	10	20.1	0.022	9.2
3rd experiment	0	18.9		
	10	22.2	0.001	17.8

[1)] *P* value was calculated using the log-rank test by comparing the survival of the untreated control group (0 μM phlorizin) to that of phlorizin-treated group (10 μM phlorizin).

[2)] % effects were calculated by (*C*-*P*)/*C**100, where *P* is the mean lifespan of phlorizin-treated group and *C* is the mean lifespan of the untreated control group.

Table S3: Effect of phlorizin and RNAi of *daf-16*/*skn-1* on Aβ-induced toxicity in *C. elegans*

	**Phlorizin (μM)**	**RNAi**	**Time when 50% of worms were paralyzed (h)**	***P* value[1)]**	**% effect[2)]**
1st experiment	0	EV	4.9		
	10	EV	7.0	< 0.001	41.9
	0	*daf-16*	4.4		
	10	*daf-16*	4.8	0.453	7.9
	0	*skn-1*	4.5		
	10	*skn-1*	4.1	0.622	-7.1
2nd experiment	0	EV	4.9		
	10	EV	6.4	0.001	29.8
	0	*daf-16*	5.3		
	10	*daf-16*	5.1	0.729	-3.8
	0	*skn-1*	5.5		
	10	*skn-1*	5.1	0.546	-7.9

[1)] *P* value was calculated using the log-rank test by comparing the rate of paralysis in the untreated control group (0 μM phlorizin) to that of phlorizin-treated group (10 μM phlorizin).

[2)] % effects were calculated by $(C-P)/C*100$, where P is the time when 50% of worms were paralyzed in phlorizin-treated group and C is the time when 50% of worms were paralyzed in the untreated control group.

Table S4: Effect of phlorizin and *skn-1* RNAi on increased mortality by HGD

	Supplementation	RNAi	Mean lifespan (d)	*P* value
1st experiment		EV	23.4	
	HGD	EV	19.4	< 0.001[1)]
	HGD + PZ	EV	21.3	0.004[2)]
		skn-1	20.1	
	HGD	*skn-1*	16.6	< 0.001[1)]
	HGD + PZ	*skn-1*	17.6	0.346[2)]
2nd experiment		EV	18.8	
	HGD	EV	12.9	< 0.001[1)]
	HGD + PZ	EV	21.0	< 0.001[2]
		skn-1	18.3	
	HGD	*skn-1*	13.1	< 0.001[1)]
	HGD + PZ	*skn-1*	13.3	0.796[2)]

1) *P* value was calculated using the log-rank test by comparing the survivals of no supplementation and HGD only with RNAi of the same gene.

2) *P* value was calculated using the log-rank test by comparing the survivals of HGD only and HGD + PZ with RNAi of the same gene.

HGD, high glucose diet (40 mM glucose); PZ, phlorizin (10 μM).

Table S5: Effect of phlorizin on degeneration of dopaminergic neurons

	Supplementation	Relative fluorescence (%)	*P* value
1st experiment		100.0 ± 5.76	
	6-OHDA	60.4 ± 5.28	< 0.001[1)]
	6-OHDA + L-DOPA	113.9 ± 7.20	< 0.001[2)]
	6-OHDA + PZ	102.9 ± 5.94	< 0.001[2)]
2nd experiment		100.0 ± 2.62	
	6-OHDA	73.6 ± 5.77	0.002[1)]
	6-OHDA + L-DOPA	99.4 ± 3.19	0.002[2)]
	6-OHDA + PZ	99.6 ± 4.72	0.004[2)]
3rd experiment		100.0 ± 3.70	
	6-OHDA	68.7 ± 3.27	< 0.001[1)]
	6-OHDA + L-DOPA	100.4 ± 4.33	< 0.001[2)]
	6-OHDA + PZ	107.1 ± 2.62	< 0.001[2)]

[1)] *P* value was calculated using the Student’s t test by comparing to the untreated control.

[2)] *P* value was calculated using the Student’s t test by comparing to 6-OHDA-treated group.

6-OHDA, 6-hydroxydopamine; L-DOPA, L-3,4-dihydroxyphenylalanine; PZ, phlorizin (10 μM).

Table S6: Effect of *daf-16* or *bec-1* knockdown on lifespan extension by phlorizin

		**Mean lifespan (d)**		
	**RNAi**	**Control**	**PZ**	***P* value[1)]**
1st experiment	*daf-16*	12.9	12.8	0.941
	bec-1	18.9	18.5	0.724
2nd experiment	*daf-16*	14.2	13.5	0.299
	bec-1	22.8	22.3	0.642
3rd experiment	*daf-16*	14.0	13.6	0.459
	bec-1	23.0	22.6	0.359

[1)] *P* value was calculated using the log-rank test by comparing the survival of the untreated control group (0 μM phlorizin) to that of phlorizin-treated group (10 μM phlorizin).

PZ, phlorizin (10 μM).
